# Associations between death anxiety and fear of illness progression or recurrence: A protocol for a systematic review and meta-analysis

**DOI:** 10.1371/journal.pone.0325176

**Published:** 2025-06-04

**Authors:** Tadgh Connery, Grazia D. Riotto, Daniel Macdonald, Rachel E. Menzies

**Affiliations:** 1 University College Cork, Cork, Ireland; 2 Menzies Anxiety Centre, Sydney, Australia; 3 University of Sydney, Sydney, Australia; Wuhan Mental Health Centre, CHINA

## Abstract

**Background:**

Fear of progression or recurrence of chronic physical illness has been associated with negative mental health outcomes across several conditions. Qualitative research suggests that the fear of death (i.e., death anxiety) may be associated with fear of illness progression or recurrence. However, a systematic evaluation of the relationship between fear of illness progression or recurrence and death anxiety is currently lacking. This protocol is for a systematic review of peer-reviewed, quantitative research examining associations between death anxiety and fear of illness progression or recurrence of chronic physical illnesses. Where possible, the strength of these associations will be tested through meta-analysis.

**Method:**

A systematic search of quantitative studies written in English will be conducted across six academic databases: MEDLINE; PsycINFO; PubMed; Web of Science; CINAHL; EMBASE. Each record will be screened for eligibility by two authors. Data extraction and quality assessment, using the Joanna Briggs Inventory Risk of Bias tool, will similarly be performed by two authors, with discrepancies being resolved through discussion and consensus with a third author, REM. Data will be synthesised narratively, according to Cochrane guidelines, by which sample characteristics, measurement tools for both death anxiety and fear of illness progression or recurrence, and associations between death anxiety and fear of illness progression or recurrence will be described. Where sufficient data are available, meta-analysis will be conducted using Comprehensive Meta-Analysis Version 4. If there are sufficient studies (*k *= 4), additional analyses may examine whether the size of the relationship differs between illness types (e.g., life-threatening vs. non-life-threatening). Gender and age may also be examined as potential moderators of the effect, based on available reported data in the studies. The protocol has been registered in PROSPERO (CRD42024583393).

**Discussion:**

This systematic review will further the understanding of how death anxiety and fear of illness progression or recurrence interact, and will help to shape future fear of illness progression or recurrence research with the aim of improving the wellbeing of individuals living with chronic physical illness.

## Introduction

Approximately one in three people globally lives with a chronic physical illness [1]. Chronic physical illness has been described as an assault on the body that threatens the integrity of self [2] and forces a reconstruction and reappraisal of one’s identity and health status [3]. Perceiving one’s illness as severe has been negatively associated with self-esteem, life satisfaction, and psychological wellbeing [4], with prevalence of chronic disease sharing a direct, positive effect with psychological distress [5]. Living with chronic physical illness brings with it many burdens, such as financial pressure, deterioration of quality of life, medication adherence, inability to work, and need for symptom control [1]. This leaves those who live with chronic disease at a higher risk of depression and anxiety, in comparison to those who do not [6,7]. Further, the relationship between chronic physical illness and mental health may be bidirectional. For example, depression has been shown to be associated with factors such as decreased metabolic control and monitoring, medication adherence, and increased hospitalisation over time [8].

While psychological outcomes like anxiety and depression are of great relevance to those affected by chronic physical illness (e.g., migraine, endometriosis) [9,10], they are not the only factors to affect these individuals. For instance, fear of cancer recurrence (FCR), concern about patients’ illness progressing or recurring [11], was found to be the most common psychological concern for individuals affected by cancer, in a systematic review of 130 articles [12]. FCR rates of up to 70% have been observed in cancer survivors [13], with meta-analytic evidence from 34 papers suggesting FCR to be negatively associated with quality of life and psychological wellbeing, and positively associated with anxiety, depression, rumination, and distress [14].

It has been proposed that cancer diagnosis engenders an existential crisis, triggering awareness of mortality, and invoking relevant defensive behaviours, such as avoidance and attempts to build meaning [15]. Indeed, existential concerns seem central to the experience of FCR. Existing qualitative research has examined the experience of FCR in 30 breast cancer survivors [16]. While those with low FCR levels spoke about practical concerns, such as treatment, disruptions to family life and finances, participants with moderate to high FCR expressed concern around death and the dying process [16]. They spoke fearfully about the prospect of cancer recurrence, worrying about potential death and the impact it could have on friends, family and colleagues, suggesting potential links between FCR and death-related concerns among those having recovered from cancer [16].

While research on FCR has largely focused on cancer, fear of progression or recurrence (FOPR) is a broader phenomenon affecting individuals with various chronic conditions. A meta-analysis of 12 studies found FOPR in various illnesses other than cancer, including coronary heart disease, stroke and Diabetes, to be positively associated with anxiety and negatively associated with quality of life [17]. Further insight to the experience of FOPR is granted by the paper’s narrative synthesis of qualitative data from over 9,000 participants across 25 studies, which similarly highlighted the relevance of FOPR across various disease types [17]. Echoing previous research examining FOPR in cancer survivors [16], death anxiety also appeared highly relevant to the experience of FOPR in illnesses other than cancer [17]. Participants voiced concerns about their awareness of death, and how their potential death would impact their families, as a result of illness progression or recurrence. These findings are further reinforced by a scoping review of 37 articles which found fear of death to be one of the seven most prominent fears associated with fear of illness progression in cardiac patients [18]. And so, despite the literature’s predominant focus on FOPR in relation to cancer, the existing literature, while limited, suggests death anxiety’s relevance FOPR in chronic, physical conditions other than cancer [17,18].

In sum, existing systematic reviews in this field have focused more broadly on the relationship between FOPR and mental health outcomes [17], and/or exclusively evaluated these outcomes in the context of cancer [14]. While emerging evidence suggests that death anxiety is relevant to FOPR in certain illnesses, a systematic evaluation of this relationship across chronic conditions is lacking. Further, whether death anxiety is uniquely related to terminal illnesses, such as cancer, or equivalently in other chronic physical illnesses has yet to be examined. Fears of death are particularly pertinent to explore, given that death anxiety has attracted growing recognition as a transdiagnostic construct, playing a key role in numerous psychopathologies [19]. This has led to burgeoning calls for treatments specifically targeting death anxiety, with evidence suggesting death anxiety can be effectively reduced through cognitive behaviour therapy interventions [20,21]. It is therefore essential to explore whether death anxiety is significantly associated with FOPR across physical illnesses. If a relationship is shown to exist between death anxiety and FOPR, this may help to identify potential new treatment targets, to reduce FOPR, and to improve mental health and wellbeing for those with chronic physical illnesses.

### Aim of the review

The review’s primary aim is to address the gaps in the current literature by narratively synthesising existing research that examines associations between death anxiety and FOPR across a range of chronic physical illnesses. If sufficient data are available, the strength of these associations will be examined using meta-analysis.

The proposed review will answer the following review questions:

1] Among adults affected by chronic physical illness (population), to what extent is FOPR (exposure) associated with death anxiety (outcome)?
2] Does the association between death anxiety and FOPR appear to differ depending on the type of chronic physical illness examined?
3] Do demographic factors, such as gender or age, moderate the relationship between death anxiety and FOPR?

### Objectives

1. The primary objectives are to narratively synthesise existing research examining associations between death anxiety and FOPR, and to test the strength of these associations using meta-analysis, where sufficient data are available.
2. Where sufficient data are available, the secondary aims are to examine potential moderators of this effect. In particular, the aims are to examine whether the association between death anxiety and FOPR differs between cancer other chronic physical illnesses, and to examine whether demographic factors such as gender and age moderate the association between death anxiety and FOPR.

## Methods and analysis

The Preferred Reporting Items for Systematic Reviews and Meta-Analyses Protocols (PRISMA-P) guidelines were followed when developing this protocol [22,23]. The protocol was registered with the International Prospective Register of Systematic Reviews (PROSPERO; CRD42024583393).

### Types of studies

The acronym PECO (Population, Exposure, Comparisons, Outcome) guided the inclusion and exclusion criteria of this systematic review and meta-analysis protocol (Table 1). Only peer-reviewed quantitative studies, including cross-sectional, experimental, quasi-experimental and intervention studies, written in the English language, will be included. Qualitative studies, policy briefs or reports, opinion papers, commentaries, editorials, news, theoretical papers, conference presentations, dissertations, literature reviews, systematic reviews and meta-analyses will be excluded. Non-English records will also be excluded. There will be no date limit placed on this search.

**Table 1:** Eligibility criteria for the systematic review and meta-analysis.

PECO Acronym	Inclusion criteria	Exclusion criteria
P	Adult participants, aged 18 years and above, affected by chronic physical illness (e.g., patients or caregivers)	Participants under the age of 18 years Participants not affected by chronic physical illness
E	Measure of FOPR	No measure of FOPR
C	Not applicable	Not applicable
O	Measure of death anxiety	No measure of death anxiety
Additional criteria	Quantitative studies, including correlational, experimental, quasi-experimental and intervention studies Studies written in the English language	Qualitative studies, policy briefs or reports, opinion papers, commentaries, editorials, news, theoretical papers, conference presentations, dissertations, literature reviews, systematic reviews and meta-analyses Non-English language articles

### Types of participants

The review will include studies that recruit adult participants (aged 18 years and above affected by chronic physical illness (population), for whom there are reported measures of FOPR (exposure) and death anxiety (outcome). Participants who report measures of FOPR with regards to chronic mental illness will be excluded.

#### Patient and public involvement.

Participants were not directly involved or recruited for this review, given that the research examines previously published data.

### Types of outcome measures

Death anxiety and FOPR, each measured by a validated scale in adult participants (aged 18 and above), will be considered primary outcome measures for this review. Studies will only be included if they include a measure of effect for an association between death anxiety and FOPR.

### Types of comparisons

Should sufficient data be available, the effect size for associations between death anxiety and FOPR will be compared between cancer and other chronic physical illnesses.

### Measures of effect

Given the diversity of measurement scales, there may be some variety in scores and outcomes identified. Thus, the frequency and prevalence of measurement scales will be noted in the data extraction sheet. Means, standard deviations, correlation coefficients and p-values will be extracted as measures of effect.

### Search method

The search strategy was developed by TC and REM. The search will be limited to studies published in English.

A systematic search of six academic databases (MEDLINE; PsycINFO; PubMed; Web of Science; CINAHL; EMBASE) will be conducted using the following search string: (“death anxi*” OR “thanatophobia” OR “fear* of death” OR “fear* about death” OR “dying anxi*” OR “death attitudes” OR “death anxiety”) AND (“anxiet*” OR “worr*” OR “fear*” OR “concern”) AND (“relapse” OR “complications” OR “recur*” OR “progress*”). TC will search full texts for the search string terms.

### Study selection

The research team (TC, GR, DM, RM) will independently screen identified records, first by title and abstract and then by full text, such that each record will be screened by two authors. Inter-rater reliability for the screening process will be calculated using the kappa coefficient. Data will be extracted using a data extraction sheet designed by TC for the purpose of the review. Two authors will independently extract relevant data. Conflicts and discrepancies will be resolved through discussion and consensus with a third author, REM. The screening process, including reasons for excluding records, will be documented using a PRISMA flow diagram.

### Data extraction process

The following details will be extracted from included papers:

- Authors and publication year
- Region
- Sample description
- Study setting
- Measurement tools for death anxiety and fear of illness progression or recurrence
- Effect sizes (e.g., correlations), p-values, means and SDs.

#### Missing data.

In cases where information is missing or incomplete, the authors will attempt to contact the study authors. Should they be unavailable, available data will be analysed as reported.

### Risk of bias assessment

The Joanna Briggs Institute Risk of Bias tools will be used to assess the quality of evidence and methodological quality of included studies. The appropriate risk of bias tool (i.e., Checklist for Analytical Cross-Sectional Studies, Checklist for Quasi-Experimental Studies, Checklist for Randomised Control Trials) will be applied to assess each included record’s quality based on its study design. Quality assessment will be conducted for each record independently by two authors. Papers will be classified as being low risk, high risk, or unclear, depending on their rating for each criterion, resolving discrepancies between them through mutual deliberation and consensus. Failing to reach consensus, REM as senior author, will resolve the discrepancy.

If data or details pertaining to a study’s procedure are missing from the report, the relevant corresponding author/co-author will be contacted in an attempt to retrieve this. Failing this, the data will be excluded from analysis, and this will be addressed in the review’s discussion section.

### Data synthesis

Adhering to Cochrane guidelines [24], a narrative synthesis will be conducted of the included papers examining associations between death anxiety and FOPR. Sample characteristics will be described and measurement tools for both death anxiety and FOPR will also be noted.

### Meta-analysis

Where at least four studies have measured both death anxiety and FOPR with the same respective scales, a meta-analysis will be conducted with the pooled results using Comprehensive Meta-Analysis. Statistical heterogeneity between the studies will be calculated using I^2^ values. An I^2^ statistic <25% will indicate low heterogeneity, an I^2^ statistic between 25–50% will indicate moderate heterogeneity and an I^2^ statistic >50% will represent high heterogeneity. A random-effects model will be used for highly heterogeneous studies (>50%) and, where the level of heterogeneity is not significant, a fixed-effects model will be applied to perform data pooling.

If there are sufficient studies (minimum *k *= 4), additional analyses may be conducted to compare the strength of association between death anxiety and FOPR across different illness types (cancer vs. other chronic physical illnesses), and to examine potential moderators of the relationship, including gender and age. For the purpose of the current meta-analysis, gender will be evaluated using data as reported within the individual included studies.

Publication bias across included studies will be assessed using the visual examination of funnel plot, the trim and fill procedure [25], and Egger’s regression test. If publication bias is identified through these procedures, a revised estimated effect size will be reported using the trim and fill procedure.

## Discussion

This study aims to systematically examine the association between death anxiety and FOPR, based on available quantitative evidence, and to calculate an effect size for this association, should sufficient data be available.

Existing qualitative research paints death anxiety as being a key part of the experience of FOPR. However, available quantitative evidence examining associations between death anxiety and FOPR has yet to be synthesised, leaving the extent of their association unclear. Strong associations between death anxiety and mental health symptomology have previously been identified [19]. Further, death anxiety has been shown to be transdiagnostic across numerous types of psychopathologies [26] and its importance in conceptualising and treating mental illness has been proposed [27]. Thus, systematically evaluating whether death anxiety is associated with FOPR will aid in understanding illness-related concerns and mental health of individuals affected by chronic physical illness, and may aid in improving their wellbeing.

A strength of this review is its intention to include all types of chronic physical illness and, if there are enough studies for inclusion, examine whether the size of the association between death anxiety and FOPR differs between illness types (e.g., life-threatening vs. non-life-threatening). Gender, age and other available demographic variables may also be tested as potential moderators of the effect.

Given the breadth of research linking both FOPR and death anxiety to negative outcomes, it is worth establishing whether a significant association exists between them. In the general population, death anxiety has been shown to be effectively reduced through cognitive behaviour therapy interventions [20]. Further, other psychological interventions, particularly those focusing on creating meaning, have been shown to significantly alleviate death anxiety in patients with advanced cancer [28,29]. Thus, if a relationship is found between death anxiety and FOPR, then targeting death anxiety may have clinical implications for improving the treatment of illness concerns and broader mental health. For example, it may pave the way for future research examining whether treating death anxiety directly leads to benefits in alleviating FOPR. In addition, should differences be found in the size of relationship between death anxiety and FOPR between cancer and other chronic physical illnesses, future research may also consider whether targeting death anxiety leads to significantly different improvements in FOPR and broader mental health as a function of disease type.

Finally, the current review will also allow for an examination of the current scope of research in this area. In doing so, it will identify certain types of illnesses that are potentially under-represented in the literature at present and pave the way for future research with benefit to further understand factors associated with FOPR and to lend itself to improving the overall wellbeing of people living with chronic physical illness.

## Supporting information

S1 File PRISMAP checklist. (DOCX)
